# Understanding the concept and importance of the health research system in Palestine: a qualitative study

**DOI:** 10.1186/s12961-018-0315-z

**Published:** 2018-06-18

**Authors:** Mohammed AlKhaldi, Yehia Abed, Constanze Pfeiffer, Saleem Haj-Yahia, Abdulsalam Alkaiyat, Marcel Tanner

**Affiliations:** 1 Swiss Tropical and Public Health Institute, Socinstr. 57, 4002 Basel, Switzerland; 2 University of Basel, Petersplatz 1, 4003 Basel, Switzerland; 3 Al-Quds University, Faculty of Public Health, Jerusalem, Palestine; 4 University Teaching Hospital, Najah National University, Nablus, Palestine; 5 Najah National University, Faculty of Medicine and Health Sciences, Nablus, Palestine; 6 Bristol University, School of Clinical Sciences, Bristol, United Kingdom

**Keywords:** Health experts, Understanding, Health research system, Palestine

## Abstract

**Background:**

The importance of a health research system (HRS), namely an instrument for developing and enabling health systems, is increasing, particularly in developing countries. Assessing the perceptions of system performers is a necessary part of system analysis, which seeks to recognize a system’s strengths and limitations aiming towards improvement. This study assesses the perceptions of policy-makers, academicians and experts regarding the HRS concept and its importance to generate insights for system strengthening. In Palestine, HRS is just emerging, helping to address the many public health-related challenges faced by the country.

**Methods:**

The study was implemented from January until July 2016, targeting three sectors, namely relevant government institutions, schools of public health, and major local and international health agencies. Data was collected through 52 in-depth interviews and six focus group discussions (FGDs) with policy-makers, academics, directors and experts. Participants and institutions were selected based on stated criteria and peer review. Data were translated, transcribed, checked and then imported to a software program (MAXQDA 12) for thematic and content analysis.

**Results:**

A total of 104 experts participated, wherein 52 were interviewed and 52 participated in the six FGDs. The HRS concept, as defined by WHO, was conceptualized differently among participants with unclear delineations between various components. Inconsistencies appeared when participants attempted to conceptualize HRS in broader contexts, though HRS goals and functions were sufficiently delineated. The majority of participants agreed that HRS correlates with notions of ‘improvement’ and recognized HRS ‘as a significant gain’. Neglect of HRS was perceived as a big loss.

**Conclusions:**

The study revealed that the level of understanding of HRS among health experts in Palestine is inadequate and not sufficiently conceptualized for its application. Findings also underlined the need to establish a central governance coordination body that promotes HRS understanding, awareness and culture as an enabler for HRS strengthening.

**Electronic supplementary material:**

The online version of this article (10.1186/s12961-018-0315-z) contains supplementary material, which is available to authorized users.

## Background

The development of health research systems (HRS) has become an international concern in recent years, particularly in low- and middle-income countries. HRS is considered a key pillar of healthcare systems (HCSs) for better health policies and equitable care [1, 2]. Research is defined in WHO’s strategy on research as “*the development of knowledge to understand health challenges*” [3] through an effective and efficient HRS to address society’s needs [4]. Further, health research (HR) is defined as “*the process for systematic collection, description, analysis, and interpretation of data that can be used to improve health*” [1]. The concept has undergone numerous refinements, including the development of a conceptual framework for National Health Research Systems (NHRSs) [5] in an attempt to correct the 10/90 gap, whereby less than 10% of global research funds are devoted to diseases that account for 90% of the global disease burden [6, 7]. In addition to establishing the Global Forum for HR to address HR gaps [6], WHO launched an HR strategy focusing on HR culture, priorities, capacity, standards and translation [3, 8].

This study adopts the WHO definition of HRS, as follows, “*The people, institutions, and activities whose primary purpose is to generate high-quality knowledge that can be used to promote, restore, and or maintain the health status of populations. It can include mechanisms adopted to encourage the utilization of research*” [9]. HRS is an emerging concept for many stakeholders, who expect to conceptualise it and realise it in practice. HRS encompasses a wide range of actors in charge of producing, consuming, managing or evaluating the system [10]. An inclusive understanding of the system concepts, importance and performance from multiple angles, incorporating the perspectives of various stakeholders, is essential [11–13].

In the Middle East, HR still suffers from lack of investment. There is a paucity of studies on perceptions of the HRS concept and importance. Several countries have not yet sufficiently examined this critical part of system understanding, nor have they assessed the performance of this system with the aim of its appropriate steering [5, 14]. Further, few countries have a formal NHRS, where building HRS is one of the challenges [11]. However, cultivating and improving an evidence-based culture is vital [15]. The Palestinian scientific research scene is unclear with regards to the lack of research orientation [16]. The present study therefore sought to demystify this ambiguity and to fill the knowledge gap in light of insufficient HRS assessments and a scarcity of literature [17], given that the WHO toolkit for HRS analysis does not address the aspect of actors' perceptions in understanding the system or assessing its performance [14, 17].

A conceptual framework designed by WHO setting the foundation of an NHRS, serves as a basis for operational analysis [18]. In this respect, various challenges have been identified, two of which are addressed in this study, namely an inadequate understanding of research and an insufficient appreciation for HRS at the political level [2, 16, 19, 20]. A deep understanding of the HRS concept and its performance is an enabling factor that could support HRS strengthening and application on the ground [10, 21]. This perspective would allow stakeholders to improve their conceptual understanding and technical potentials alike, and allow the achievement of good health outcomes and equity. Overcoming the inadequate understanding of HR is considered one of the study’s motives [18]. Further, a better understanding of the HRS concept and its performance might also lead to improvements in other HRS components such as governance, capacity, policy, priorities and stakeholders.

Such an understanding could lead to a sustainable HRS, whereby HR culture and knowledge is promoted among stakeholders and HR is embedded as a philosophy, based on shared conceptualisation [22, 23] and institutionalisation practices. A shared HR concept would contribute to decision-making and policy development based on evidence. The present study is part of a larger investigative research project that holistically explores Palestinian HRS components. Three diverse and relevant sectors in Palestine have been purposively targeted – government, academia (public health schools), and health non-governmental organisations (NGOs) and international agencies. As a logical first step, this study provides an overview of the perceptions of health policy-makers, academics and experts involved in HRS with regards to their understanding of the HRS concept. Such an overview can provide a foundation on which to build and reinforce a HR culture and strengthen other HRS pillars. Specifically, the paper aims to (1) assess the level of understanding among health policy-makers, academics and experts regarding the definition and conceptualisation of the HRS concept and to capture their perceptions of its goals and functions; and to (2) assess the perceptions of HRS stakeholders in Palestine and the associated gains and losses of adopting or dispensing with HRS in the country.

## Methods

### Design

Descriptive situation analysis was developed based on data collected through qualitative methods, in-depth interviews (IDIs) and focus group discussions (FGDs) to inductively investigate the perceptions of HRS stakeholders in Palestine. System thinking and comprehensive perspective approaches were adopted, both of which are helpful to understand and map system dynamics through a wide-range approach [22, 24]. Furthermore, the study used the NHRS framework for system analysis as it is both sensitive to limited resource settings and enables local experience and understanding to be built and serve as a starting point for NHRS improvement [12, 14]. The study design suits the complexity of HCS and the HR environment, and help us to understand the research topic from numerous perspectives [25]. The study was conducted in the West Bank and Gaza Strip of the Palestinian territories, which are geographically segregated (Additional file 1: Supplement 1). The study was conducted from January until July 2016. We targeted institutions across three sectors (illustrated in Additional file 1: Supplement 2) as follows:

1. Six government bodies: Ministries of Health, Higher Education, Finance and Planning, Palestinian Legislative Council, Palestinian Medical Council, and Palestinian Central Bureau of Statistics.
2. The academic sector, namely health and medical faculties in 10 major universities and colleges in Palestine, and an expert from Lebanon who has intensively researched and written about this subject.
3. The local and international NGOs, namely 10 international NGOs and 11 local Palestinian NGOs.

#### Sampling and data collection

A purposeful approach to sampling was used. The initial list of potential participants across the three sectors was prepared based on the knowledge and experience of the first author, a Palestinian, who has worked more than 9 years in the three sectors and has a background in public health. Participants were allocated to one of two groups, wherein 52 political key informants participated in IDIs and 52 participants with technical expertise were assigned to FGDs, without double participation. Expert consultations and rigorous peer reviews were performed to attain sample representation. Only then were the participant lists merged into one final list. To ensure knowledge saturation level, active participation and adequate representation, mixed purposive sampling was achieved through four strategies. First, criterion sampling made it possible to select participants who could provide specific information on certain study topics. Second, critical case sampling targeted experts to give critical and factual information on the topics under investigation. Third, snowball sampling determined other suitable participants, as we were aware that there were likely other key informants that were not known to us at the outset of the study. Finally, homogenous sampling brought together participants from a similar background and with similar experience [26]. Inclusion and exclusion criteria were established to guide the selection process clearly (Additional file 1: Supplement 3).

The study was designed to diversify participants based on their level of knowledge, experience and positions, and their levels of involvement in HRS across the three sectors. The principal investigator phoned and emailed potential participants and provided them with a copy of the study information sheet. Participants who did not respond to the initial contact received another call and email a couple of weeks thereafter. In total, 104 experts from across the sectors agreed to participate, either in IDIs or FGDs. Prospective participants received the full agenda and discussion outlines in advance via email, followed a few days later by an invitation. Selection equilibrium of participants was achieved between both included areas (West Bank and Gaza Strip). Participants from executive political and front management levels of targeted HRS institutions were assigned to IDIs, while participants from middle technical and management level were appointed to FGDs. The grouping was intended to obtain diverse reflections and a comprehensive understanding.

For both IDIs and FGDs, open-ended questions were drawn up, assembled and adapted according to the principles laid out in the relevant literature [1, 3, 9, 14, 18, 27], and can be found in Additional file 1: Supplement 4 (4a for IDIs and 4b for FGDs). Both instruments focused on five themes, namely (1) HRS conceptualisation and its importance (the focus this study); (2) stakeholder satisfaction with HRS performance; (3) governance, policy and finance; (4) stakeholder analysis, HRS capacities and research priorities in Palestine; and (5) HRS challenges and insights for strengthening.

To appraise trustworthiness and credibility, questions were discussed among the research team as well as with the support of international scientists and local experts in Palestine. The questions were piloted in five IDIs and in one FGD to check their clarity and to provide a basis for cross-checking subsequent responses. Building on the pilot, we revised both questionnaire instruments.

A total of 45 IDIs were conducted face-to-face and seven by Skype call due to movement restrictions in the field. IDI duration ranged between 45 and 60 minutes. Eighteen interviewees were academics from different health schools, 20 interviewees were representatives from the six government bodies, and 19 were experts from 10 local and five international NGOs. The six sectoral FGDs were performed three each in the West Bank and the Gaza Strip, with only one FGD for each sector in both areas. Each FGD lasted approximately 90 minutes and included 6–10 participants. IDIs and FGDs were conducted in Arabic by the first author, a middle-aged, male Palestinian. A trained research team coordinated and managed all data-collection field work guided by the principal investigator.

### Data analysis

Data collected from IDIs and FGDs were audio-recorded. The discussions were held in Arabic and were simultaneously translated and transcribed in English into MS word sheets, which were then revised for precision, checked and cleaned for accuracy. The data were subject to both thematic and content analyses [28]. Themes and codes were deductively established based on the conceptual framework developed by the relevant HRS literature. Field notes were kept and used during data collection and analysis. All transcripts were imported into the software MAXQDA 12 (VERBI GmbH, Berlin). Subsequently, the first author created codes and read each transcript, line by line. Data were then displayed in a particular matrix according to the respective themes and codes. Selected data were reviewed and discussed carefully with the team to identify patterns.

## Results

### Sociodemographic characteristics of participants

Of the 115 experts from 38 institutions across the three sectors invited to participate, 104 agreed and actively responded to both methods of inquiry, while 11 invitees declined due to scheduling conflicts. As HR is conceptually broad [29], participants came from diverse professional backgrounds and areas of expertise. They were selected to represent the three disciplinary categories of (1) public health, (2) medical and biomedical, and (3) economic and political fields. Public health covered various areas, such as health management, finance policy, nursing, community and mental health, child and women’s health, nutrition, social policy, school health and education, non-communicable diseases, epidemiology, and water, sanitation and environment. Medical and biomedical fields covered pharmaceutical, biology and laboratory, biochemistry, and clinical medical and surgical fields. Participants also represented other disciplines, for instance, economic, political and legislative such as Ministry of Finance and Planning and the Palestinian Legislative Council.

Table 1 describes the characteristics of 52 participants from the three sectors, 38 of which were male. The majority held PhD degrees, most had more than 20 years of experience, particularly in NGOs, but a few had less than 10 years. Eighteen academics, 10 of whom were senior faculty members, represented eight academic institutions. Of the 19 participants representing 15 NGOs (10 local and five international), eight were executive directors while the rest were heads of offices, departments and programmes. Fifteen participants represented five government institutions, four from Gaza and 11 from WB, where the central government sits. Seven government participants served in advanced-level leadership roles with respect to policy- and decision-making, while the rest were directors or heads of departments.

**Table 1:** Characteristics of in-depth interview participants

Sector	Ch.ch.	Age			Sex		Educational level			Years of experience			Participants per location				
Acad.		30–50	51–60	> 60	F	M	BA/Dip	MA	PhD	< 10	10–20	> 20	West Bank	Gaza Strip	Lebanon	Jordan	Egypt
		7	7	4	5	13			18		11	7	10	7	1		
		Leadership positions											No. of institutions vs. locations				
		VP	Dean		VD		HRD		Associate Professor		Assistant Professor		4	3	1		
		1	5		2		2		6		2		Participants: 18, Institutions: 8				
Gov.		30–50	51–60	> 60	F	M	BA/Dip	MA	PhD	< 10	10–20	> 20	No. participants and institutions vs. locations (alike)				
		5	7	3	3	12	4	6	5	1	6	8	West Bank	Gaza Strip	Lebanon	Jordan	Egypt
		Leadership positions											11	4			
		FM/DM	NHM		GD		Director		HD				Participants: 15 Institutions: 5 and 9 departments				
		3	4		1		3		4								
NGOs		30–50	51–60	> 60	F	M	BA/Dip	MA	PhD	< 10	10–20	> 20	No. of participants vs locations				
		11	8		6	13	1	13	5	3	4	12	West Bank	Gaza Strip	Lebanon	Jordan	Egypt
		Leadership positions											10	7		1	1
		ED	Director		HO		CO		PO				Participants: 19 Institutions: 15, 10 local NGOs and 5 international NGOs				
		5	3		2		4		5								

*ch.ch.*: socio-demographic characteristics

Sectors: Acd: academic, Gov: government, NGOs: non-governmental organisations

Sex: F: female, M: male

Education: BA/Dip: bachelor and diploma, MA: master, Ph.D.: doctor of philosophy

Position: VP: vice president, D: dean, VD: vice dean, HRD: head of the research department, FM/DM: the former minister or deputy minister, NCM: national council member, GD: general director, HD: head of department, ED: executive director, HO: head of the office, CO: chief officer, PO: programme officer

Table 2 describes the six FGDs with 52 participants from 24 institutions, performed in parallel for each sector. Two FGDs targeted 14 academics; three were female and most had more than 10 years’ experience. The third and fourth FGDs included 20 government policy- and decision-makers; five were female and participants mainly had post-graduate degrees in public health; 13 had 10–20 years of experience in various high-level positions. The fifth and sixth FGDs comprised 18 experts from six local and seven international NGOs; seven were female and, notably, 15 held a master degree in public health. Most had more than 10 years experience; five experts served in an executive directorate, while the rest were in the same executive level but with more operational and technical duties.

**Table 2:** Characteristics of focus group discussion participants

Sector	Ch.ch	Age			Sex		Educational level			Years of experience			Total		
2 Acad. FGDs		30–40	41–50	> 50	F	M	BA/Dip	MA	PhD	< 10	10–20	> 20	Participants (14)		Institutions (8)
													West Bank FGDs	Gaza Strip FGDs	
			7	7	3	11			14	4	6	4	6	8	
		Leadership position			D		FP			Associate Professor			Assistant Professor		
					1		1			5			7		
2 Gov. FGDs		30–40	41–50	> 50	F	M	BA/Dip	MA	PhD	< 10	10–20	> 20	Participants (20)		(18): 4 institutions 14 departments
													West Bank FGDs	Gaza Strip FGDs	
		2	8	10	5	15	1	10	9		13	7	12	8	
		Leadership position			NCM		GD			D			HD		
					1		8			5			5		
2 NGO FGDs		30–40	41–50	> 50	F	M	BA/Dip	MA	PhD	< 10	10–20	> 20	Participants (18)		(13): 6 LNGO 7 INGO
													West Bank FGDs	Gaza Strip FGDs	
		3	12	3	7	11	3	15	2		8	8	10	8	
		Leadership position			ED				PM				SO		
					5				8				5		

*ch.ch.*: socio-demographic characteristics

FGDs: focus group discussions

Sectors: Acd: academic, Gov: government, NGOs: includes local and international non-governmental organizations

Sex: F: female, M: male

Education: BA/Dip: bachelor and diploma, MA: master, Ph.D.: doctor of philosophy

Position: D: dean, FP: full professor, NCM: national council member, GD: general director, D: director, HD: head of the department, ED: executive director, PM: programme manager, SO: senior officer

Interviewing the system actors was a basic step towards capturing the overall understanding of HRS. Building a sustainable system fundamentally requires a basis that enables an HR culture and an understanding of its actors. Only then is it possible to embark on strengthening other system pillars. Misconceptualising a system may result in confusion and negatively affect its performance by creating duplications and inefficiencies, and affect the credibility of the research produced within this system [23]. For that purpose, the study began with the primary theme of conceptualisation and an overall understanding of HRS among participants. The study contained two relevant sub-themes, namely (1) the overall understanding of the definition of HRS, where interviewees and FGD participants were asked to delineate the concept and describe how they realised its goals and functions; and (2) the thoughts evoked by the mention of HRS, and the perceived gains and losses associated with its application or disuse.

### Conceptualising HRS, its goals and functions

Participants across the three sectors were asked in both IDIs and FGDs to define HRS as a concept and to describe its goals and functions. Not all of the participants’ responses were fully consistent with the adopted definition. Answers to this question revealed obvious variations among the experts’ conceptualisation levels. Responses were extremely wide ranging. Most participants defined HR rather than HRS, but a few gave accounts corresponding to the WHO definition. Differences in conceptualisation may relate to their divergent backgrounds and expertise, and their knowledge and awareness of the HRS concept, goals and functions.

The majority agreed that HR is merely a scientific process and tool. They considered HR an indispensable element to reinforce the Palestinian health system and to improve health based on evidence. Participants sufficiently recognized HR goals by stating that it generates knowledge that can be used for community benefits. However, a small number of respondents clearly conceptualised HRS and appraised its goals and functions. The views of eight respondents from the three sectors were almost consistent with the adopted definition wherein HRS is an integrated system that includes different institutions dedicated to producing scientific research on specific health phenomena, to find solutions that feed the decision-making level required to formulate suitable policies. Many experts agreed that this system supports the health sector and its functions, guiding health needs, evaluating results and planning health interventions to reach a suitable health condition.

The following selective key quotes show the differences among the three sectors with regards to the definition and conceptualisation of HRS. Selected responses illustrated the above findings. One stated that, “*… A basic and essential system runs the HR and its policies including the priorities and methods of research. It also means using the research to support the HCS in Palestine as one unit along with other resources for better health services*”*.* [Academic Expert 12].

A WHO expert took a wider approach to HR, delineating concerned people and system scope: “*… HR is a very wide domain and includes both basic and applied research. People concerned with this system comprise basic researchers (basic clinical), epidemiologists …etc.*” [WHO Expert 4]. A different senior WHO participant, relying on his experience and national figures, emphasized that Palestine recognizes the meaning of HRS better than other countries: “*…In Palestine, we have a better understanding of HR, more than any other Arab country*”. [WHO Senior Expert 3].

A senior government planner expressed that the HRS is a network consisting of a spectrum of people, policies and resources to tackle major health problems. Correspondingly, he reported its goals and functions: “*… It is a network of interested people in HR, who work together toward mobilising the needed resources to strengthen health. As we know, HR is the cornerstone for the improvement and development of HCS services. It aims to reflect the community’s real needs, and it is not a matter of using it as a tool to get a master or a PhD degree. The goal of HRS is to address the serious health problem to promote the health of the population*” [Senior Gov. Expert 12].

The vast majority of IDI perceptions were combined, where there was a common pattern among experts to describe HR in general rather than HRS specifically. In other words, most responses went in the same direction, with some defining HR as a static scientific approach that aims to identify the problems of development in health. Others indicated that HR is a scientific tool or process involving a range of organised activities that aim to examine health problems and to produce evidence that improves health through supporting informed decisions. One academic conceptualised HR as follows: “*…As a scientific approach, it seeks for development and improvement within the health sector aspects. It aims to explore the actual health problem by analysing it, in order to conclude an effective solution, followed by integrating the outcomes and solutions inside the decision-making process*” [Academic Expert 1].

There was also a quite general perception from other academics who did not define a system. One added that HR is an assessment that focuses on a certain health problem to produce evidence to be used in the decision-making process. Likewise, a local NGO expert explained that HR is a process within an organised system and linked to decision-making to reflect the impact on the community: “*…It is related to a scientific process that comes up with results that give a chance to the decision-makers to integrate these results in developing the community on different levels. It is a process comprising organised steps that could lead to developing the community*” [NGO Expert 6]. Supporting this perspective, one expert from an international NGO working in Palestine revealed that the concept of HRS is any research related to public health yielding credible scientific evidence to solve a particular problem.

Very few offered a consistent definition where they broadly defined the HRS. Three academics described it as an observation, which stems from community needs and generates evidence that contributes to solutions. Other experts defined it in different ways – an academic stated that it is “*everything that is concerned with human health*”, while another said that it is “*kinds of activities*”. NGO participants expressed it as “*Understanding the health problem*”, “*all research that targets health*”, “*prevailing problem for which there is a need to investigate its causes*” and “*to produce results for problems in the health system*”. A very frequent response was that HRS is “*anything that is related to public health*”. One government expert loosely delineated it as “*trying to find out more about certain health-related aspects*”, while another from the same sector said that HRS is “*using numbers to analyse data and describe the reality of problem*”. A national council member asserted that the concept of HRS differs from one culture and country to another. One academic prefaced their definition by claiming that the definition of HR is not well known and applied, while a second denied the existence of any system for HR, stating:

“*…We do not have a system. I am against the idea of saying a system where the HCS in Palestine does not exist; there are some fragments, which we can try to unite to make a national system for a number of reasons. First, we are under occupation and this is a great challenge that creates many obstacles in front of us. Secondly, we do not have enough financial resources to improve our research unless there is some kind of international organisation to finance research. However, these organisations usually control and steer the research according to their agenda. In our research institutes, we sometimes challenge aid providers because they force their own agenda against our national needs and priorities. For example, we have problems like non-communicable diseases and they are not targeted properly. We have a huge problem in meeting the needs of the society in HR. The main reason, after all, is the international organisation and their goals along with the lack of attention to the importance of HR*” [Academic Expert 16]

Regarding HRS goals, another common pattern emerged among expert responses, wherein they each mentioned at least one goal. The most frequent HRS goals identified were to produce knowledge to develop the HCS and the community, and to find evidence to improve health services and solutions for health problems. Regarding HRS functions, most of the experts were not familiar with the main system tasks as adopted by WHO, namely those of (1) stewardship, such as setting vision, priorities and ethical standards, coordination, and HRS monitoring and evaluating; (2) securing finance; (3) creating and sustaining human and physical resources; and (4) procuring validated outputs and translating them into practice [9, 18]. No response was consistent with the WHO conceptual framework for HRS functions. They roughly delineated these thoughts on system functions in broad and everyday language. For instance, no one mentioned the stewardship concept and very few indicated the importance of vision and priorities, or stated the need to measure and evaluate the system. Many experts mentioned system financing and resources and capacity-building, but those functions were not declared in their definitions.

### Experts’ perceptions of HRS

This second part of the study is dedicated to the expert’s perceptions that emerged when HRS was mentioned. It also addresses their key thoughts related to the gains reaped when HRS is adopted and the losses when it is dispensed with. This too contributed to capturing the overall understanding of HRS among leading persons and institutions. Most interviewees from all sectors responded to this section (Table 3) and agreed that mentioning HRS raised a variety of thoughts (themes) that essentially reflect the HRS components. These thoughts/themes, ordered sequentially according to frequency and weight, were (1) HCS and healthcare improvement; (2) resources; (3) burden of disease and population epidemiological status; (4) HRS vision and regulatory system; (5) HR culture; (6) political interest and issues related to academia, policy-making and planning; (7) development issues and priorities; (8) cooperation; (9) statistics; and (10) rationalisation. This means that experts thought actively and productively in delineating relevant thoughts, where all declared issues were inherently related to the system’s elements. Furthermore, most saw that HRS is a true developmental model that contributes to the improvement and progress of the health sector.

**Table 3:** Main responses of in-depth interview participants when ‘health research system’ was mentioned

No.	Theme	Quotes to support the theme	No.	Theme	Quotes to support the theme
1.	HS and healthcare improvement	- Concrete evidence could improve health work - A system concerned with health services development - HRS offers a continuous improvement of the health system - A system contributes to healthcare improvement and provided it properly - I think about improving the health system in Palestine - HRS promotes healthcare effectiveness and efficiency - Reminds me of problems, e.g. management problems in HRS - Mention the fruitful results of research to be invested in provided services - Improves the theories and tools which we need to deliver a better health system - Relates to services provided and their efficiency and effectiveness - Links with the effectiveness of medical intervention - Quality of health services - Brings hope and willingness to develop the health system via well-developed HRS	6.	Policy-making, planning, problem-solving	- We recall poor policy-making and planning based on evidence - Health planning and problem-solving - A precious chance to solve problems scientifically to be used in policy-making - All health interventions which must be based on scientific research - The link between research, policy and intervention does not exist
2.	Resources and funds	- A serious lack of national resources linked to funding - There are no funds or donations for HRS - We think about even financial support as a major obstacle, we have knowledge and qualities available, but the problem is in funding - Due to the limitation of resources, a need to link the HRS with research efficiency, effectiveness and decision-making - A lack of researchers in some specific areas of research - We are working on the theoretical aspect, not the practice because we do not have laboratories or research centers to sponsor operational health research - Severe lack of both financial and human resources - Financial support constitutes an obstacle for NGOs to conduct research	7.	Development linked, strategies, priorities and society needs	- A precious opportunity to be linked with development and vice versa - To study the needs of society, we do not want to do research just for research, it has to be based on social priorities and needs - We do not have priorities in HRS - Identifying priorities and strategies for HRS - We do studies essentially based on national priorities
3.	Epidemiology status, BODs, NCDs, communicable, population, mortalities	- I recall the burden of disease such as NCDs - I consider tuberculosis, AIDS, neonatal mortalities - Directly connected with epidemiology - Research related to the diseases that threaten the life of Palestinians - Remembering topics such as communicable diseases and NCDs - Gazan people who live in a highly populated limited area - Documenting BODs among Palestinian refugees - Determines the health problems and provides solutions to be tackled - We have many problems, e.g. neoplasm diseases	8.	Association and cooperation	- Disassociation between institutions that produce and use research - Networking, cooperation and coordination - HRS means that researchers have to be allied with each other - Health research is the mutual language between scientists and links us with the international community
4.	Vision, system, or regulation and evaluation	- A system involves all actors, make the health system integrative without duplication - The absence of a system or governing body, and no well-known entity responsible for HRS processes - One unified body adopts and supervises HRS, but we do not have it - HRS needs to be controlled and supervised by professionals, not by unprofessional people, which also evaluate its activities - We do not have a clear vision for tackling the health problems - A need for health research to evaluate the skills of health workers, managers, policy-makers or researchers - Finding ways to improve HRS, identify and overcome challenges	9.	Statistics and data	- There are no accurate statistics in Palestine - There is a clear inconsistency on data completeness and availability in Palestine
5.	Culture, interest and academia related	- Health research in our region is sporadically controlled by the donor and conducted particularly for short-term and rarely for long-term projects - Previous attitudes on HRS were neglected, but nowadays there is interest, HRS is affected culturally in its progress, and there is a lack of required attention - We do not have enough attention for HRS, which is seasonal - Related to academia, operational research is undeveloped, most of the research done in public health schools which are neglected and utilised - Many universities and institutions are interested in HRS, the orientation status is more developed than before	10.	Rationalisation	- Rationalisation, research helps us to identify the best options along with cost-effectiveness and efficiency; without any agenda, the research process is illogical

*BODs* Burden of diseases, *HRS* health system research, *NCD* non-communicable diseases

Responses in FGDs were largely consistent with those from the IDIs. Most centered on the purpose of HRS, improving health and lifestyle through finding solutions to prevent diseases. Respondents in FGDs agreed that HR is fragile, neglected and devalued. Severe lack of awareness about it was the most frequent concern. Other responses pointed to improper documentation and a plethora of unused data, or that HR is not linked with our lives and institutional activities. Academic expert 1 emphasized that “*HR is inherently personal-driven for self-development desires only, not vision-driven or initiated by national or institutional orientation. Also, the aspects of regulatory body and allocated budget and resources are questionable*”, representing the most common response among all sectors. In general, experts bemoaned the absence of integration and emphasized the importance of cooperation between institutions and universities. Experts in government FGDs called for increased knowledge production by strengthening HRS pillars; other expressed that HRS systematically scrutinises the national indicators and causes of disease to build useful policies. An academic described the concept as an “*unrealistic political logo and discourse*”, but two others noted that it linked with public health. NGO experts believed that HRS is not aligned with actual needs and its outputs are unlikely to be put into action. Another NGO expert stated that HR is commonly descriptive rather than operational and experimental.

Subsequently, the degree of importance or unimportance experts give to HRS was assessed. Experts were questioned on what is gained and or lost from using or not using HRS. Table 4 show the responses from both tools, which were mostly focused on seven sub-codes under the gains–loses codes, revealing how HRS both positively and adversely affects (1) population; (2) HRS and HCS; (3) planning, policy and development; (4) technical services; (5) priorities and needs; (6) evidence and decisions; and (7) resources. The majority fully agreed that we would gain a lot from adopting HRS. The most frequent responses were associated with the sub-code ‘technical aspects’, indicating that HRS is seen as improving healthcare and offering proper management. The next most frequent code was ‘population’, meaning that HRS contributes to promoting health by combating risks and finding solutions. Moreover, codes ‘HRS and HCS’ and ‘evidence and decisions’ were to some extent saturated by experts, indicating that HRS strengthens HCS and plays a major role in successfully planning and developing strategies that lead to health development. Another less frequent sub-code was ‘feeding credible evidence to the decision-making levels’, and even less frequently expressed were the sub-codes ‘indicating that a system contributes to prioritizing our needs’ and ‘promotes optimal uses of resources’. One of the most comprehensive and prominent quotes was expressed by a government policy-maker, “*HCS and HRS are considered two sides of the same coin, and a driving force to steer the wheel of health work. HRS supports and evaluates HCS pillars and updates its staff knowledge and potentials*”.

**Table 4:** Responses from in-depth interviews and focus group discussions on what we gain and lose from health research systems

Theme	Code	Sub-code	Quotes from sectors		
			Government	Academic	NGOs
HRS Understanding	Gains	Population	Identify hidden issues to find solutions, identify causes and risk factors of disease and formulate plans to eliminate them	Prevent health problems, improve health status, guide health research to address social needs, tackle the problems facing patient and environment	Understand national indicators and problems, produce knowledge to reduce BODs, address community threats, all health challenges will be tackled, and improve people conditions
		HRS-HCS	Health system and HRS are two sides of the same coin, steer the wheel of health work, support health system pillars, evaluate the system and update staff knowledge	Clear path to see where we are heading, HCS improvement, reform health system policies and evaluate interventions	Determine HCS strengths and gaps, developed a health system
		Planning, policy, development	Proper planning, develop effective health policies based on evidence	The essence of development devoted to solving health dilemmas based on evidence and scientific approach, solving problems by converting its results into policies, policies based on evidence	Gives guidelines for strategies, changing policies
		Technical-services	No repetitions of programs, equity,	Collaboration with great outputs and gives an advantage to the interdisciplinary spirit, improve certain medications, public health and health services promoted, better health services and our goals will be achieved	Better health care, evaluate programs, minimize duplicated studies, increase the quality of care, organized research actions, management-based evidence, care effectiveness, efficiency, quality, care cost containment, effective interventions, improve health care, technology and knowledge, regulated duties and satisfied clients and decision makers
		Priorities-needs	Generate priorities reflect needs, determine our real needs		Prioritize needs, meeting our needs
		Evidences-decisions	Perfect solutions based on concrete evidence, findings to be considered in policies	Add value to rational and evidence-based decisions, better effectiveness in decisions and efficiency, having evidence-based policies and actions	Reliable evidence-based answers for use by policymakers, evidence-based information, help decision making, new policies, expanding knowledge, evidence on problems, results from available resources
		Resources		Better resources, a guidance to harness limited resources effectively	Save resources
	Loses	Population	Economic collapse, unknown risk factors, and causes	Will not get a good health status for people, many unsolved problems	Increase economic and social burden of disease, the high prevalence of diseases and disabilities
		HRS- HCS	HS problems cannot be solved and tackling them will be random and improvisational, losing everything within health system pillars	We cannot dispense of HRS, the value and importance of research will be declined, failure will be our fate due toHRS missing which is the essence of HCS, we would not lose if research is done appropriately	Cannot improve health system
		Planning, policy, development	Different visions and agendas, random policies, the picture will be unclear as we work in the darkroom which affects negatively on health	Lack of policies based on research, we cannot measure, predict and change in the health field	We cannot improve and evaluate health sector
		Technical-services	No cooperation and each Institute works separately, repeating our efforts without progress, research conducted unsystematically-randomly which will not reflect reality, research duplication, losing connections	Will lose harmonic work among partners, health care will be disorganized, ineffective and inefficient	Ineffective management, evidence to measure the quality of care will be lost, duplication of studies and activities, health care will be duplicated and cost ineffective, quality of care cannot be improved
		Priorities-needs		Cannot determine priorities and needs	Inability to identify serious problems, losing solutions for problems
		Evidences-decisions	Limited knowledge and poor application, accidental and random decisions	HRS outputs unexploited in decision making then unconsidered actions, actions taken without evidence, inaccurate and ineffective decisions, a total disaster for decision makers	
		Resources	Losing human resources to be updated with knowledge and skill	Missing resources, wasting efforts, time and resources, disperse our efforts	Ineffective resources allocation, wasting human resources, resources can be lost if research did not add value, wasting efforts, inability to control research resources and meet the society needs

Likewise, under the code ‘loses’, responses could be subdivided into seven comparable sub-codes. All experts agreed in saying “*we will lose nothing*”, and were mainly concerned about technical aspects, namely that without HRS, there is a risk of uncoordinated healthcare and duplicate, mismanaged, ineffective and inefficient research activity. The second most frequent sub-code was ‘HCS’, meaning that, in the absence of HRS, HCS will not be improved and problems will remain unsolved. The next sub-codes were ‘policy and planning’, which can only succeed with HRS, and otherwise consist of different visions and an unclear picture. The ‘resources’ sub-code received responses to the point of saturation, whereby the majority expressed that a lack of HRS could lead to constant resource waste and limited workforce knowledge. Other less expressed sub-codes were ‘priorities’ and ‘needs’ (meaning that neither would be determined). One government official noted, “...*missing HRS creates different visions and agendas in the health field, the scene of this field will be unclear, as we are working in a dark room, which reflects negative effects on health interventions*”. In addition, an academic member said, “*...we will not achieve a good health status for people and there will be many unsolved health problems*”.

## Discussion

The approach used herein offered a deep understanding of the importance and performance of HRS for developing a well-functioning national research system [4, 5]. We focused on exploring the understanding of the HRS concept, its goals and functions, and on determining the key concerns related to HRS in terms of what is gained and lost when it is adopted or neglected.

Study participants across all sectors were highly responsive to the thematic questions. The importance of HRS, its role in the health field and its potential for strengthening this system was acknowledged by most. However, participants stated that such a system and its enabling environment have not yet been established. IDI and FGD responses were largely consistent with each other, without a prominent difference of perceptions among the three sectors. The overall understanding of HRS and its relevant components, as defined by WHO, was inadequate, particularly the concept of HRS, where descriptions from most of the policy-makers, academics and NGO experts were not fully aligned with the WHO definition adopted in 2004 [17, 18].

A lack of system understanding meant that the majority of experts were only familiar with and sufficiently aware of ‘health research’ as a broader concept, as demonstrated by a study on defining research to improve HCS [29]. Our study intersected with a previous study that found that the largest deficits were in understanding HR from a systems perspective [18]. Similarly, deficient levels of awareness and lack of appreciation for an HR culture are problematic factors contributing to system underperformance [16]. In other words, expert delineations on HRS do not align with the WHO’s definition adopted in this study. Differences in perception regarding the HRS definition were observed across the sectors, where academia defined it from a pure knowledge and scientific perspective, while government and NGOs delineated it from a more practical point of view. This proves that most of the definitions were not framed in a harmonized and integrative way and were not defined from a system perspective. There are different explanations for this finding, including the complexity of the system and its constituents [29], the concept of HRS still being emergent [30], the weakness of the curriculum in schools of public health to cover HRS concepts and of its application not being adequately endorsed, and the lack of leadership concern and unsupportive environment around developing a HR culture and practice. Aggravating the situation is the absence of a political will – HR is not on the agenda of the Palestinian government and this was found to be one of the main obstacles to better system performance [31]. These factors are exacerbated by poor incentives, political difficulties, conflicting priorities and an unappreciated research culture [11, 16, 19, 20, 32, 33]. Findings do show, however, that experts have conceptualised the goals of HRS to some extent, with responses aligning with those of another relevant study [18]. The experts highlighted that the main goal of HRS is to generate knowledge for use in policies to improve health and the community. Other studies assert that HR is needed to attain the Sustainable Development Goals [34]; this response is compatible with the definition of health policy and systems research [35]. Further, the conceptual framework of HRS functions as outlined by the WHO initiative [14] was not appropriately recognised by experts. Among the functions commonly identified by all respondents were guiding health needs, evaluating the results and planning health actions to achieve suitable health conditions.

The policy-makers, academics and NGO experts we met were enthusiastic in their responses, and provided many key thoughts and perceptions about HRS, raising some critical issues. The diversity and volume of thoughts and insights captured contribute to a better understanding of the Palestinian context as it relates to HRS. During their conceptualisation, respondents recognised 10 themes, half of which could be categorised as development ideas and half as the difficulties associated with HRS. Most of the identified themes largely coincided with findings from a particular study that discussed these themes as HRS challenges [3, 36]. These were HCS and service improvement; insufficient resources; population health problems and burden of disease; absence of a regulating system and vision; a donor-driven research agenda rather than a culture-driven or academically based agenda; and policy, planning and decision-making. These six issues were mentioned most frequently, while other less frequent themes addressed the failure to adopt HRS as a development tool and to identify its priorities based on community needs and a general lack of cooperation and connection. The last two themes were data unavailability and accuracy and HRS as the best option for cost-rationalisation [11].

The study also pointed to strengthening factors of HRS in practice, as perceived by experts. HRS was seen as essentially contributing to health status improvement through finding solutions to prevent chronic diseases and to improve lifestyle. This perspective has been emphasised by another recent study [37]. Thus far, HR is still fragile, neglected, devalued and relatively unknown or understood, which can be attributed to weak political will and concern to officially adopt HRS as a strategy and to take the necessary steps to strengthen it. Without political interest, it is not possible to develop most HRS components [36]. A national unified strategy that endorses actual community health needs is a critical in Palestine. This should be accomplished by a national policy developed through wide consultation and consensus with stakeholders and by allocating financial resources and incentives to strengthen HRS across all sectors [13]. At the same time, international organisations can be motivated and become a catalyst for success. Our findings regarding the main obstacles and strengthening actions correspond to conclusions drawn by others [11, 38], namely that those challenges are not only prevalent in Palestine as a low-resource and unstable country, but also throughout the Middle East region in general.

The concept of HRS raised some pivotal issues. We found that there has been an improvement in research productivity in Palestine, as in the Eastern Mediterranean region overall [11]. Yet, the quality of research produced by many Palestinian institutions has not yet reached a satisfactory level [39]. What is worrying, however, is that these efforts inherently stem from personal desires for self-development, rather than being motivated by or performed according to a visionary institutional and national agenda. This reinforces the hypothesis that HR is inefficient and ineffective, where most experts were dissatisfied with its system performance. There is an absence of national vision and dynamic regulation, as well as a lack of integration and cooperation among interested stakeholders; this conclusion coincides with relevant evidence from WHO [40]. Correspondingly, system underperformance is the result of non-participatory coordination among HRS stakeholders [11, 38].

The study found that HRS is generally appreciated, but it is unfortunately not realised on the ground. Most of the experts elaborated numerous fundamental thoughts, which were reflected as themes, during their conceptualisation. The study assumed that the gains of HRS identified indicated its importance, whereby the goals and functions of HRS were similarly characterised. In contrast, the stated losses from HRS were assumed to indicate a lack of importance. The overall perceptions indicated that HRS was seen as important, particularly toward guaranteeing HCS improvements, proper management and evidence for decisions. HRS was also seen as contributing to the overall development of resource-poor countries by helping to address its problems, support successful planning and policy formation, encourage optimal use of resources and, ultimately, identify priorities and needs [37, 41]. In contrast, neglect of HRS was seen to lead to enormous technical losses in the form of random and uncoordinated healthcare actions, research duplication and inefficiency, and ineffectiveness and mismanagement. Moreover, at the system level, neglecting HRS is seen as leading to unimproved HRS and HCS and unsolved problems, unsuccessful planning and policies, different visions, continuous wasting of resources, and shallowness of priorities and needs. Evidently, ensuring that we do not lose sight of the goals and importance of HR would allow us to see the significant benefits of research in everyday practice [18, 42].

The study has some limitations. Relevant literature, particularly studies investigating the perceptions of HRS players, was not adequately implemented, likely due to poor political attention and culture towards HR. Furthermore, the research team was limited in its access to other relevant institutions and experts due to movement restrictions. Further, time limitations prevented the involvement of more leadership levels across sectors, but this was addressed through the assistance of key experts who facilitated intensive communication and cooperation of their senior partners across institutions. Some IDIs and FGDs were shorter than the expected time, and some questions were not sufficiently answered, either due to limited knowledge, practice or time constraints.

## Conclusion

HRS is increasingly appreciated globally as a substantial pillar of the HCS structure to improve health [11]. Hence, there is a need to functionally link research to HCS. To achieve this, a shared understanding of HRS concepts is an important first step towards developing this system. By realizing these concepts, relevant actors are likely to increase their commitment and involvement, which may ultimately lead to better outcomes in research management, production and utilisation. This requires performers to conceptualise the system, which may also create a pioneering orientation among health policy-makers to coordinate their actions with each other and with other development sectors. Varying definitions or vague conceptualisations may cause confusion and become an obstacle to progress [23].

The study assessed the perceptions of policy-makers, academics and experts to emphasise the importance of addressing their conceptual understanding pattern of the HRS, which is a basic requirement in system analysis towards system strengthening [9]. The overall understanding of Palestinian policy-makers and experts on the HRS concept is strikingly inadequate, yet the importance of the system and its benefits were certainly recognised. Therefore, we conclude by calling for a serious move to increase understanding and awareness of HRS [11, 16, 20].

The concept of HR is more understandable among respondents than the HRS, as evidenced by the ambiguous and imprecise conceptualisation of the system and its components compared to the WHO definition. There are significant HR attempts among respective researchers and policy-makers. However, the process of increasing awareness and understanding remains sluggish, which suggests that HR is not sufficiently internalised or embedded in the culture. Another interpretation is that HR is not performed in a systematic way based on a collective vision, but rather spontaneous and individually, which may have limited yields.

Many of the system components, such as governance, policy, finance, knowledge sharing and coordination, are not practically applied in the Palestinian health sector. The importance of the system is fully appreciated by most of the experts as a major gain, while neglecting it is recognised as a great loss. However, the goals and functions of the system, as delineated [4, 8, 18], were sufficiently recognised. Moreover, the concept of HRS correlates with improvement approaches [9], where most respondents linked HRS to developmental ideas (e.g. HCS improvement, meeting societal needs, effective policies and planning, correct decisions, tackling health problems, and resource conservation). In contrast, others more readily associated HRS with the difficulties or challenges facing the system.

The analytical approach used in this study, based on stakeholders’ perceptions, could be a useful analytical framework and basis for broader system analysis. Stakeholders’ perceptions of other system components could be investigated, and indeed this approach has not been adequately addressed in most of the respective attempts to analyse the system [5, 9, 14, 18]. A qualitative investigation to assess conceptualisation patterns could reflect the real internal mentality and state of thinking among those involved in the system. Additionally, the present analytical approach may also be used as a complementary and operational assessment method alongside other known approaches, leading to a better and inclusive understanding of HRS [9, 18]. More empirical research is needed to more clearly identify the reasons behind the apparent lack of awareness and knowledge about the HRS concept and its uses and applications.

To conclude, enhancing the level of HRS knowledge and culture among researchers and policy-makers is indispensable for HRS strengthening. Knowing the stakeholders’ conceptualisation patterns contributes to enabling system practices and applications, creating opportunities for successfully institutionalisation of appropriate system components within the Palestinian HCS. In other words, a well-conceptualised system makes research a practical and essential tool for analysing and solving health problems. If this issue is successfully addressed politically, HR and HRS in all sectors could become reality rather than rhetoric. To embed the HRS concept and its components in research culture and to make it applicable by policy-makers and experts, HCS decision-makers should adopt the following strategies:

1. Inform the various decision-making levels, through a national workshop, of the key findings of this study and explain the importance of strengthening the concept and application of HRS within the Palestinian HCS.
2. Strengthen national political will and decisions (e.g. among senior Ministry of Health staff with international support from WHO) to support cooperative efforts towards enabling the HRS concept as an integral element of the national health strategy.
3. Invite all stakeholders for a strategic dialogue to formulate a national HR policy, leading to a national body integrated into the Palestinian HCS to take responsibility for creating appropriate institutional mechanisms to perform system functions.
4. Enable this body to steer all relevant stakeholders and apply the HRS framework, including forming research leadership, resources, priorities, roles and coordination. At the forefront of four central operational steps is promoting the awareness and culture of the HRS concept among research producers and users, and the importance of its application – the first building block towards successfully implementing the action framework through:
  Establishing a national institutional policy that focuses on raising HRS awareness among health system policy-makers and professionals. This can be achieved through intensive education and capacity-building programmes and on-the-job training activities to develop their HRS potential, with an incentive mechanism to encourage them.
  Redeveloping the curricula of academic programmes of public health schools to become more research oriented, including HRS components into curricula.
  Promoting local and international knowledge exchange programmes, provided by WHO and Council on Health Research for Development (COHRED), through platforms, policy dialogues, publications, workshops and meetings on the HRS concept, goals, functions and applications.
  Eventually establishing alliances and mutual partnerships, targeting HCS stakeholders, to expand their knowledge and understanding of HRS conceptual frameworks, related HRS concepts, approaches, applications and utilisation, through dynamic knowledge dissemination channels.

## Additional file

Additional file 1: **Supplement 1.** Palestine map. **Supplement 2.** List of targeted institutions across the government, academic universities and local and international NGOs who working in Palestine. **Supplement 3.** Selection criteria for selected study institutions and participants. **Supplement 4.** The study instruments (IDIs and FGDs). (DOCX 66 kb)

## Abbreviations

FGDs: focus group discussions
HCS: healthcare system
HR: health research
HRS: health research system
IDIs: in-depth interviews
NGOs: non-governmental organizations
NHRS: national health research system
